# Effects of monosaccharides including rare sugars on proliferation of *Entamoeba histolytica* trophozoites *in vitro*

**DOI:** 10.3389/fmolb.2023.1288470

**Published:** 2023-12-08

**Authors:** Kentaro Kato, Mitsumasa Miura, Hiroshi Tachibana, Ikuko Tsukamoto

**Affiliations:** ^1^ Department of Eco-epidemiology, Institute of Tropical Medicine (NEKKEN), Nagasaki University, Nagasaki, Japan; ^2^ School of Tropical Medicine and Global Health, Nagasaki University, Nagasaki, Japan; ^3^ Department of Parasitology, Tokai University School of Medicine, Isehara, Japan; ^4^ Department of Pharmaco-bio-informatics, Faculty of Medicine, Kagawa University, Kita-gun, Japan

**Keywords:** *Entamoeba histolytica*, amitochondriate, trophozoite, monosaccharide, rare sugar, proliferation

## Abstract

*Entamoeba histolytica* is a parasitic protozoan with roles in pathogenicity of intestinal amoebiasis. *E. histolytica* trophozoites lack functional mitochondria and their energy production depends mostly on glycolysis. D-Glucose has a pivotal role in this process and trophozoites store this sugar as glycogen in glycogen granules. Rare sugars, which are defined as sugars present in nature in limited amounts, are of interest as natural low-calorie sweeteners for improving physical conditions of humans. One such rare sugar, D-allose, can be absorbed by a sodium-dependent glucose cotransporter as a substitute for D-glucose, and some rare sugars are known to inhibit growth of cancer cells, *Caenorhabditis elegans* and *Tritrichomonas foetus*. Based on these observations, we examined the effects of rare sugars on growth of *E. histolytica* trophozoites, together with those of D-galactose and D-fructose. The results indicate that treatment with D-allose or D-psicose (D-allulose) alone inhibits proliferation of *E. histolytica* trophozoites, but that these sugars enhance proliferation of trophozoites in the presence of D-glucose or D-galactose. The trophozoites could take up D-glucose and D-galactose, but not D-fructose, D-allose or D-psicose. Cell sizes of the trophozoites also differed depending on the culture medium.

## 1 Introduction

*Entamoeba histolytica* (*E. histolytica*) causes amoebiasis and an estimated 50 million cases of dysentery, colitis and extraintestinal abscesses, resulting in more than 55,000 deaths annually (Lozano et al., 2012). *E. histolytica* infects humans as cysts by the feco-oral route with food or water and also by sexual transmission. The trophozoites excyst in the small intestine, migrate to the large intestine and cause amoebiasis through destruction of the mucus layer and epithelial cells. Among *E. histolytica* infected cases in humans, 90% are self-limiting and asymptomatic. However, in a few cases, trophozoites cause liver abscess and encephalopathy, which may be fatal (Haque et al., 2003). Metronidazole is the drug of choice for this infection, despite its known side effects (Roe, 1977). However, trophozoites remain in the intestines in 40%–60% of treated cases (Petri, 2003). Moreover, metronidazole-resistant *E. histolytica* trophozoites may be induced *in vitro* (Samarawickrema et al., 1997), suggesting that the resistant strain can appear naturally with metronidazole therapy. Indeed, metronidazole-resistant *E. histolytica* clinical isolates have been reported recently (Bansal et al., 2004; Dario, 2008; Iyer et al., 2017; Victoria-Hernández et al., 2020). Furthermore, metronidazole given orally is absorbed almost completely, with bioavailability >90% for tablets (Lamp et al., 1999) and may not have sufficient effects on *E. histolytica* trophozoites reside in the large intestine. Therefore, a safe alternative class of drugs is required that will not induce emerging resistant *E. histolytica* strains and will reside in the large intestine to eliminate the trophozoites. If the drugs can decrease the number of *E. histolytica* trophozoites in the gastrointestinal tract, the number of expelled cysts will also decrease, and thereby, the transmission will be suppressed. The protozoan parasite lacks typical mitochondria (amitochondriate) and solely relies on glycolysis for adenosine triphosphate (ATP) generation (Ali and Nozaki, 2007; Jones and Ingram-Smith, 2014). Therefore, D-glucose has a pivotal role in the life cycle of the parasite and stereoisomers of D-glucose are candidates for prevention or treatment of *E. histolytica* infection by affecting the glycolysis pathway.

Sugar-free foods that do not contain D-glucose may have an effect on transmission of *E. histolytica* trophozoites in humans, but meals without sugars become tasteless. Thus, rare sugars have attracted attention as natural low-calorie sweeteners for improving physical conditions in humans (Ahmed et al., 2022). These sugars serve as sweeteners, do not spoil the flavor of the food, and are hardly metabolized (Smith et al., 2022). Seventy to 80% of orally administered D-psicose will be absorbed from gastrointestinal tracts, but the rest will reside in the large intestine (Whistler et al., 1974; Tsukamoto et al., 2014). Some rare sugars have also been shown to have biological effects; for example, D-allose inhibits growth of human cancer cell lines (Noguchi et al., 2016); other rare sugars affect the growth (Sato et al., 2008; Sakoguchi et al., 2016a; Sakoguchi et al., 2016b) and lifespan (Shintani et al., 2017; Shintani et al., 2019) of *Caenorhabditis elegans*; and D-allose and D-psicose both inhibit growth of *Tritrichomonas foetus* and reinforce the action of metronidazole on the parasite (Harada et al., 2012). Therefore, rare sugars may also have anti-amoebic functions. In this study, we examined the potential of monosaccharides, including rare sugars, to serve as anti-amoebic substances *in vitro*.

## 2 Materials and methods

### 2.1 Sugars

D-Glucose, D-galactose and D-fructose were purchased from FUJIFILM Wako Pure Chemical Corp. (Osaka, Japan). D-Allose and D-psicose (D-allulose) were purchased from Tokyo Chemical Industry Co., Ltd. (Tokyo Japan). To avoid confusion with D-allose, we use the name “D-psicose”, instead of D-allulose, in this study, even though the International Rare Sugar Society (ISRS) recommends usage of D-allulose (https://www.isrs.kagawa-u.ac.jp/RSS/image/AlluloseNAME-2.pdf). Structures of the sugars are shown in Supplementary Figure S1A.

### 2.2 Cell culture

*E. histolytica* trophozoites (HM-1: IMSS cl6) were cultured axenically in TYI-S-33 medium supplemented with 51.4 mM D-glucose, 15% adult bovine serum (B9433, Sigma-Aldrich, St. Louis, MO), 1.6% penicillin/streptomycin solution (168–23191, FUJIFILM Wako Pure Chemical Corp., Osaka, Japan) and 2.3% vitamin mix solution (58980C, Sigma-Aldrich, St. Louis, MO) at 37°C. In modified media, D-galactose, D-allose, D-fructose or D-psicose (51.4 mM each) was added instead of D-glucose. Medium without sugars was used as no sugar medium. In some studies, 25.7 mM Sugar A and 25.7 mM Sugar B half-and-half media (Sugar A/Sugar B media) were used.

### 2.3 Time course assay

*E. histolytica* trophozoites were seeded at 2.0–3.0 × 10^4^ cells/mL/vial in D-glucose medium in 1-mL glass vials (Maruemu Corp., Osaka, Japan) and cultured for 24 h at 37°C to allow attachment to the wall of the vials and grow. Trophozoites in one of the vials were then harvested for cell counting. D-Glucose medium in other vials with trophozoites was removed and changed to 1 mL of fresh medium containing D-glucose, D-galactose, D-allose, D-fructose or D-psicose, and the trophozoites were cultured for an additional 24, 48, 72 and 96 h at 37°C. In some studies, 1 mL of half-and-half medium was used. Trophozoites in 1-mL media without sugars were also cultured for the same time periods. A photograph of the culture system and the study protocol are shown in Supplementary Figure S1B, C, respectively. Data were collected from 5 independent experiments.

### 2.4 Number and viability of *Entamoeba histolytica* trophozoites

After trophozoites were cultured for 24, 48, 72 and 96 h at 37°C, the cells were detached from vials on ice and the number of cells was counted using Bürker-Türk C-chips (NanoEntek, Seoul, Korea). The viability of the cells was determined by Trypan blue exclusion test. Viability of trophozoites was calculated as: Viability (%) = (live trophozoites/total trophozoites) × 100. Cell images were captured using an EVOS-XL microscope (ThermoFisher Scientific, Supplementary Figure S2B). Five independent studies were conducted.

### 2.5 Size of *Entamoeba histolytica* trophozoites

Images of trophozoites in each culture condition were taken after 48-h culture at 37°C and the major axis of the trophozoites was measured. Data are summarized as box plots. Forward Scatter histograms with 2,500 events were obtained using a Gallios™ Flow Cytometer (Beckman Coulter, Inc., CA, United States) for comparison of the cell sizes. The histograms were analyzed using Kaluza Analysis software ver. 2.1 (Beckman Coulter, Inc., CA, United States, Supplementary Figure S2C).

### 2.6 Measurement of monosaccharides remaining in culture media of *Entamoeba histolytica* trophozoites

Monosaccharides remaining in media after 96-h culture at 37°C were assayed and compared with amounts in 0-h culture media. The assay was conducted using high-performance anion-exchange chromatography with pulsed amperometric detection (HPAE-PAD), as previously described with slight modifications (Kuzawa et al., 2019). A Dionex ICS300 system equipped with a CarboPac PA1 column (4 × 250 mm) (Dionex, Tokyo; current ThermoFisher) was used. After centrifuging at 2,000 rpm for 5 min, supernatants were deproteinized by mixing with a 4-fold volume of MeOH. Samples were then filtered (0.45 µm) and diluted 10 times with 80% MeOH after centrifugation at 10,000 rpm for 10 min. The applied sample volume for HPLC was 25 μL; mobile phase: 1 mL/min, 10 mM NaOH aq. (0–35 min), 200 mM NaOH aq. (35–45 min); column temperature 35°C. Concentrations of monosaccharides in culture media were determined from the peak area on HPLC charts (Supplementary Figure S3) referring to that in standard solutions.

### 2.7 Detection of reactive oxygen species (ROS)

*E. histolytica* trophozoites were seeded at 1 × 10^4^ cells/200 μ1/well in D-glucose medium in a glass-bottom 96-well plate (No. 655891, Greiner bio-one) and were cultured for 24 h at 37 °C. Then D-glucose medium was removed and changed to 200 μ1 of fresh medium containing D-glucose, D-galactose, D-allose, D-fructose or D-psicose, and the trophozoites were cultured for an additional 24 h at 37°C. Highly Sensitive DCFH-DA Dye in ROS assay kit (Dojindo) was added to each well with 1,000 times dilution and the trophozoites were further incubated for 1.5 h at 37°C. ROS production in the trophozoites were observed under a Nikon Eclipse Ts2-FL microscope equipped with a 470 nm C-LED filtercube (Ex. 450–490 nm, Em. 507–562 nm). The fluorescence histograms (Ex. 488 nm, Em. 525 nm) of the trophozoites were obtained using a Gallios™ Flow Cytometer (Beckman Coulter, Inc., CA, United States) and the histograms were analyzed using Kaluza Analysis software ver. 2.1 (Beckman Coulter, Inc., CA, United States). The data are shown in Supplementary Figure S4.

### 2.8 Statistical analysis

Cell numbers and viability of trophozoites cultured in sugar media were compared with those of trophozoites cultured in no sugar medium by ANOVA with a Dunnett test. The size of trophozoites was compared with that of trophozoites cultured in D-glucose medium by ANOVA with a Dunnett test. *p* < 0.05 was considered significant.

## 3 Results

### 3.1 Proliferation of *Entamoeba histolytica* trophozoites cultured in monosaccharide media

**FIGURE 1:**
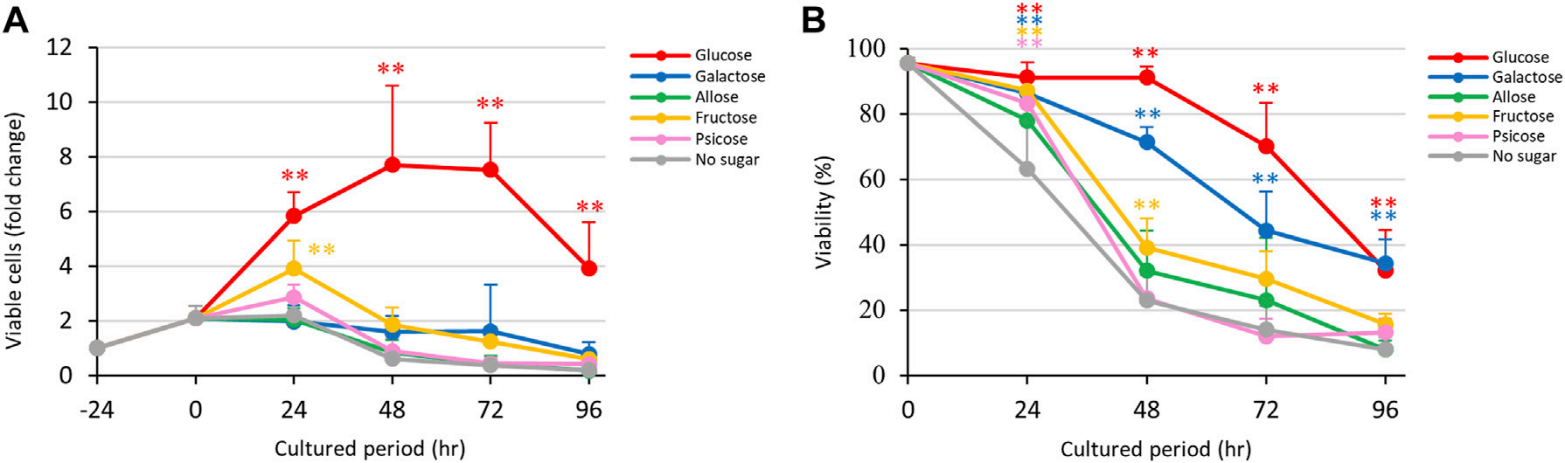
Effects of monosaccharide media on proliferation and viability of *E. histolytica* trophozoites. **(A)** Effects of media on growth of trophozoites. **(B)** Effects of media on viability of trophozoites. *p < 0.05 and **p < 0.01 vs no sugar medium (ANOVA with a Dunnett test).

*E. histolytica* trophozoites in 1-mL vials grew normally in pre-incubation for 24 h (Figure 1A). After a change to fresh medium supplemented with D-glucose, other sugars or without sugars, the trophozoites started to proliferate differently. Those cultured in D-glucose medium grew exponentially until 48 h, but then the number of viable trophozoites decreased after 72 h. This was due to overgrowth of the trophozoites in the 1-mL glass vials. Among media containing stereoisomers of D-aldose (D-glucose, D-galactose and D-allose), the growth of trophozoites in D-allose medium was similar to that in no sugar medium (Figure 1A). D-Galactose medium detached the trophozoites from the vial walls (Supplementary Figure S2A) and suppressed their growth, but the number of live trophozoites was maintained over 72 h. Culture media supplemented with D-fructose or D-psicose showed similar effects on growth, except that the number of live trophozoites increased by 24 h and then gradually decreased thereafter in D-fructose medium (Figure 1A). The number of trophozoites in these media reached the same levels at 96 h as that in no sugar medium. An overlay of the growth curves indicated a clear difference in the effect of D-galactose on growth of trophozoites at 24 h compared to that of D-fructose. Both rare sugar media (D-allose and D-psicose) showed effects on growth that were similar to that of no sugar medium.

### 3.2 Viability of *Entamoeba histolytica* trophozoites cultured in monosaccharide media

Viabilities of *E. histolytica* trophozoites in different culture media are shown in Figure 1B. Trophozoites in D-glucose medium showed about 90% viability until 48 h, about 70% viability at 72 h, and about 30% viability at 96 h. Among the D-aldose stereoisomers (D-glucose, D-galactose and D-allose), D-allose medium produced the lowest viability, especially after 48 h, and the effect was similar to that with no sugar medium. In contrast, D-galactose medium produced a moderate decrease in viability. D-Glucose and D-galactose media had significantly different effects on cell viabilities of trophozoites compared with no sugar medium. D-Ketose stereoisomer (D-fructose and D-psicose) media showed the same trend as no sugar medium, except the D-fructose had a slightly lower effect on viability. D-Psicose medium had a similar effect on trophozoite viability as that of no sugar medium. In summary, D-allose, D-fructose and D-psicose had effects on the viability of *E. histolytica* trophozoites that were similar to those of no sugar medium.

Time courses of fold changes of total, dead and viable *E. histolytica* trophozoites in different culture media are shown in Supplementary Figure S5A. The total number of cells increased over the culture period in D-glucose medium, but viable trophozoites decreased after 72 h of culture. The total number of trophozoites peaked between 24 h and 72 h in culture with D-galactose, D-allose and no sugar media, and decreased after 48 h in D-fructose and D-psicose media. Among the sugar media, D-allose medium showed a similar trend to that with no sugar medium.

### 3.3 Size of *Entamoeba histolytica* trophozoites cultured in monosaccharide media

**FIGURE 2:**
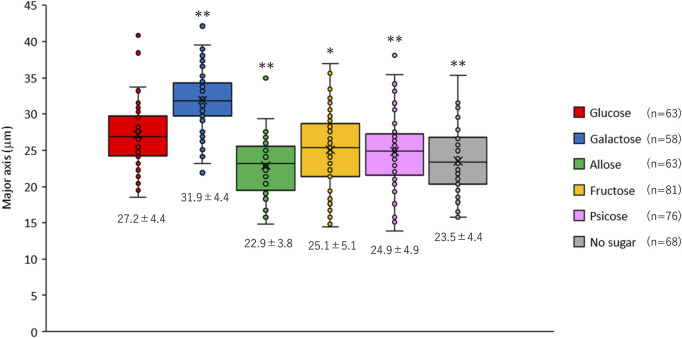
Size of *Entamoeba histolytica* trophozoites after 48 h culture in different culture media. The major axis of live trophozoites was measured using images taken under a microscope (Supplementary Figure S2B). Data are shown as box plots. The number under each box is the mean (μm) ± standard deviation. *p < 0.05 and **p < 0.01 vs D-glucose media (ANOVA with a Dunnett test).

While observing the *E. histolytica* trophozoites, we noticed that the size of trophozoites differed among the conditions (Supplementary Figures S2A, B). Interestingly, surviving trophozoites could not attach to the wall of the vials and accumulated at the bottom in culture in D-galactose medium (Supplementary Figure S2A). Most of the accumulated trophozoites at the bottom of the vials in other media were dead cells (data not shown). To evaluate the difference in size of trophozoites in different culture media, the major axis of viable trophozoites was measured (Figure 2). The size of trophozoites in D-galactose media was significantly larger than that in D-glucose medium. In contrast, the sizes of trophozoites cultured in D-allose, D-fructose and D-psicose media were significantly smaller than that in D-glucose medium. The size of trophozoites in rare sugars and D-fructose media were similar to that in no sugar medium. These results were objectively confirmed by overlaying forward scatter plots obtained by FACS analyses (Supplementary Figure S2C).

### 3.4 Proliferation of *Entamoeba histolytica* trophozoites cultured in half-and-half media

**FIGURE 3:**
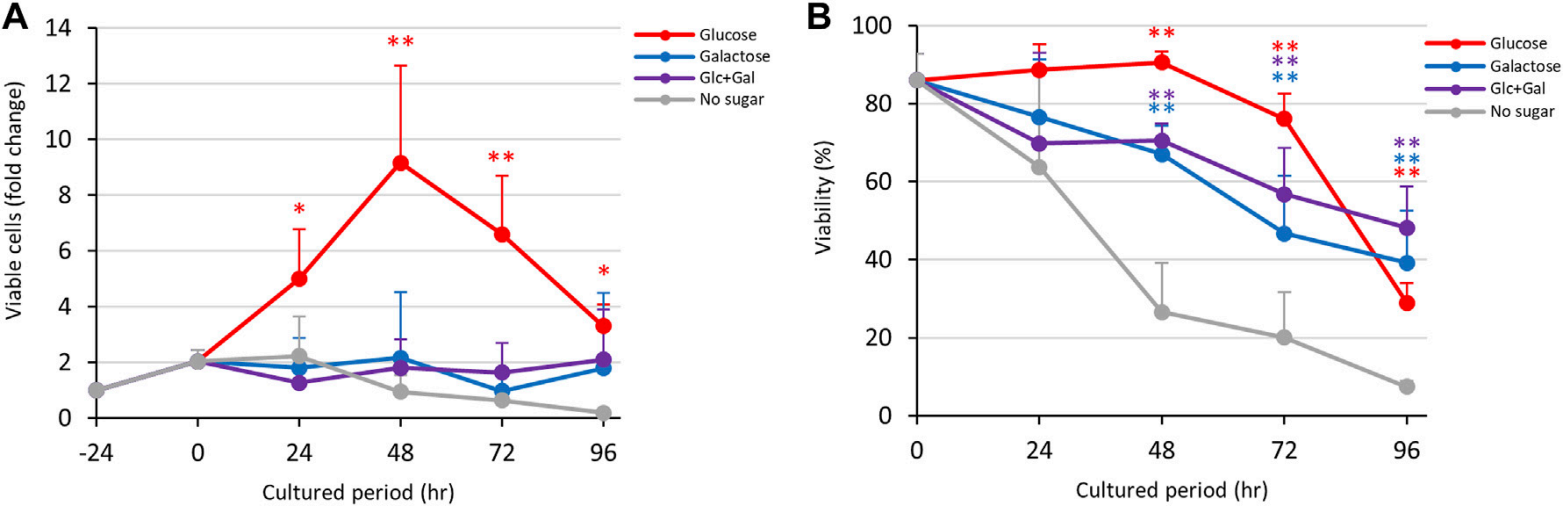
Effects of Glc/Gal medium on proliferation and viability of *E. histolytica* trophozoites. **(A)** Effects of media on growth of trophozoites. **(B)** Effects of media on viability of trophozoites. *p < 0.05 and **p < 0.01 vs no sugar medium (ANOVA with a Dunnett test).

As mentioned above (Figure 1), D-glucose promoted growth while D-galactose maintained viability of trophozoites, even though the trophozoites detached from the vial walls in D-galactose medium (Supplementary Figure S2A). To examine which sugar had the most favorable effect on proliferation, we cultured the trophozoites in D-glucose/D-galactose half-and-half medium (Glc/Gal medium) (Figure 3). To our surprise, the trophozoites in this medium could not grow, in contrast to the behavior in D-glucose medium (Figure 3A). The viability in Glc/Gal medium was comparable to that in D-galactose medium throughout the culture period (Figure 3B). As in D-galactose medium, the number of viable cells was sustained in Glc/Gal medium throughout the period without increasing the total number of cells (Supplementary Figure S5B), indicating that the effects of D-galactose in the medium were more important than those of D-glucose.

**FIGURE 4:**
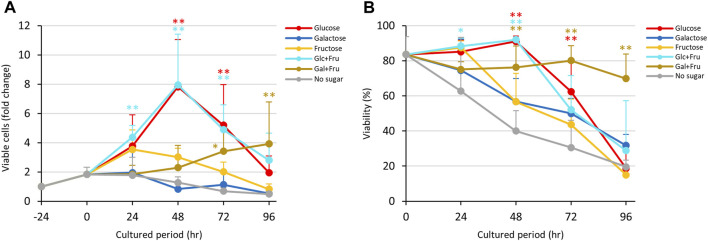
Effects of Glc/Fru and Gal/Fru media on proliferation and viability of *E. histolytica* trophozoites. **(A)** Effects of media on growth of trophozoites. **(B)** Effects of media on viability of trophozoites. *p < 0.05 and **p < 0.01 vs no sugar medium (ANOVA with a Dunnett test).

The results in Figure 1 indicate that D-glucose and D-fructose accelerated proliferation of *E. histolytica* trophozoites at 24 h, while D-fructose could not maintain proliferation and the number of viable trophozoites decreased after 48 h. In contrast, D-galactose suppressed proliferation and sustained the viability and number of viable trophozoites (Figure 3). To examine whether D-fructose can enhance the effects of D-glucose and overcome the effects of D-galactose on proliferation, we cultured the trophozoites in D-glucose/D-fructose (Glc/Fru) and D-galactose/D-fructose (Gal/Fru) half-and-half media (Figure 4). Trophozoites proliferated with the same trend in Glc/Fru and D-glucose media, even if the concentration of D-glucose in Glc/Fru medium was half that in D-glucose medium (Figure 4A). In contrast, trophozoites proliferated in D-fructose medium for 24 h, but the number of viable cells decreased after 48 h, showing the same results as those in Figure 1. Interestingly, trophozoites gradually proliferated in Gal/Fru medium, which did not occur in Glc/Gal medium (Figure 3A). Furthermore, viability in Gal/Fru medium was maintained at about 70% even after 96 h of culture, in contrast with other culture conditions (Figure 4B). The total number of trophozoites was higher in Glc/Fru medium than in D-glucose medium at 96 h (Supplementary Figure S5C). The total cell counts gradually increased in Gal/Fru medium, but this count was only sustained or was reduced in D-galactose or D-fructose media (Supplementary Figure S5C).

### 3.5 Proliferation of *Entamoeba histolytica* trophozoites cultured with rare sugars

**FIGURE 5:**
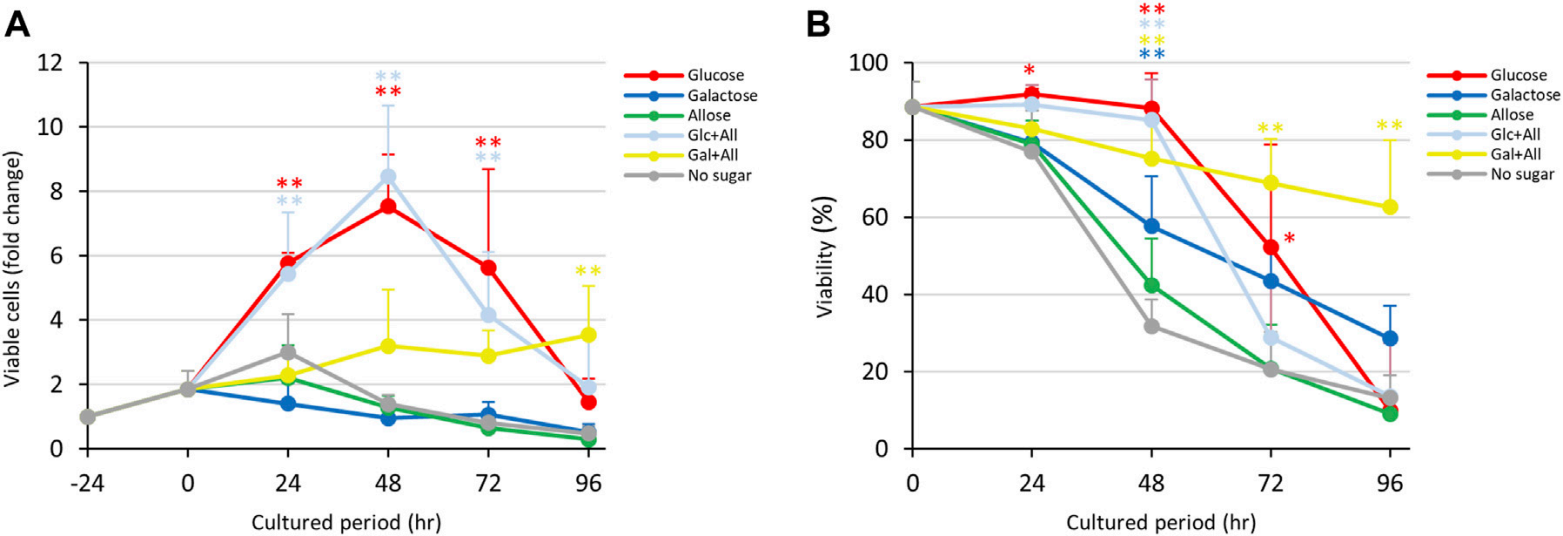
Effects of Glc/All and Gal/All media on proliferation and viability of *E. histolytica* trophozoites. **(A)** Effects of media on growth of trophozoites. **(B)** Effects of media on viability of trophozoites. *p < 0.05 and **p < 0.01 vs no sugar medium (ANOVA with a Dunnett test).

**FIGURE 6:**
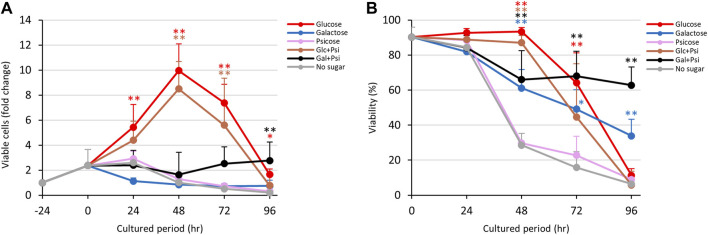
Effects of Glc/Psi and Gal/Psi media on proliferation and viability of *E. histolytica* trophozoites. **(A)** Effects of media on growth of trophozoites. **(B)** Effects of media on viability of trophozoites. *p < 0.05 and **p < 0.01 vs no sugar medium (ANOVA with a Dunnett test).

As shown in Figure 1, D-allose and D-psicose suppressed proliferation and viability of *E. histolytica* trophozoites. To examine whether these rare sugars block the effects of D-glucose and D-galactose, trophozoites were cultured in D-glucose/D-allose (Glc/All), D-galactose/D-allose (Gal/All), D-glucose/D-psicose (Glc/Psi) or D-galactose/D-psicose (Gal/Psi) half-and-half media (Figure 5; Figure 6).

Contrary to our expectations, trophozoites in Glc/All medium proliferated with the same trend as that with D-glucose, with similar viabilities throughout the culture period (Figures 5A,B). Gradual proliferation of trophozoites occurred in Gal/All medium with sustained viability, similarly to trophozoites cultured in Gal/Fru medium (Figure 4; Figure 5). The total number of trophozoites in Glc/All medium was slightly higher than that with D-glucose at 96 h; this was also observed for trophozoites in Glc/Fru medium (Supplementary Figure S5C, D). The gradual increase in the total number of trophozoites in Gal/All medium over time showed the same tendency as that in Gal/Fru medium (Figure 4; Figure 5).

In Glc/Psi and Gal/Psi media, proliferation of trophozoites was comparable with those with Glc/All and Gal/All, respectively (Figure 6). The number of viable trophozoites in Glc/Psi medium showed a similar trend to that in D-glucose medium (Figure 6A). Viability of trophozoites in D-galactose medium gradually reduced, while that in Gal/Psi medium more gradually reduced and was sustained at 60%–70% after 48 h (Figure 6B). The total number of trophozoites was higher in Glc/Psi medium compared with that in D-glucose medium, and the number of viable trophozoites in Gal/Psi medium gradually increased after 72 h of culture (Figure 6A and Supplementary Figure S5E).

Collectively, D-fructose, D-allose and D-psicose enhanced proliferation of *E. histolytica* trophozoites with D-glucose and supported gradual proliferation of trophozoites with D-galactose.

### 3.6 Measurements of remaining amounts of monosaccharides after 96-h culture in each medium

**FIGURE 7:**
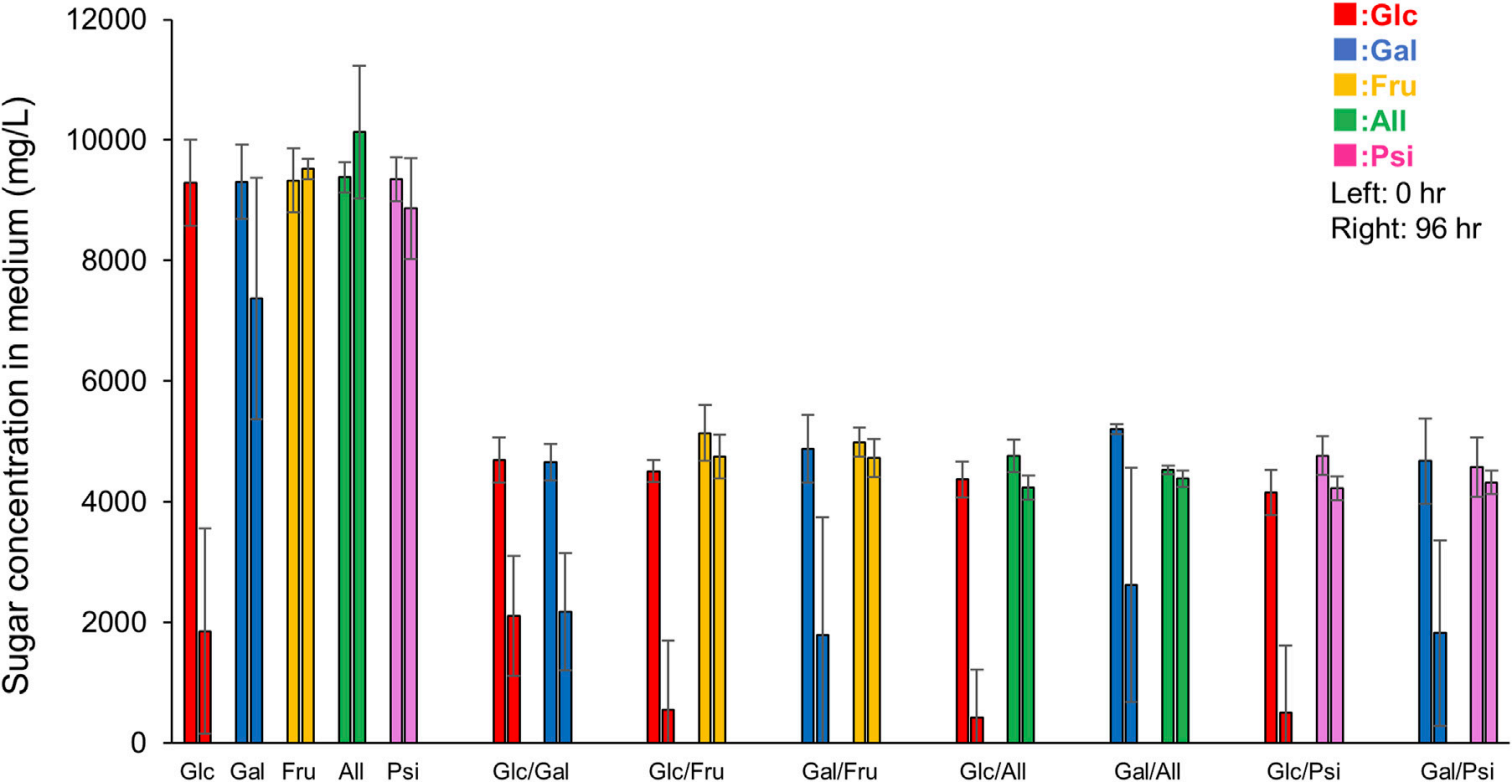
Remaining monosaccharides in 96-h culture media. Peak areas on HPLC charts (Supplementary Figure S3) were used to determine remaining concentrations of monosaccharides in 0-h or 96-h culture media referring to that of standard solutions. Data are averages of at least 5 independent studies (mean ± standard deviation). Sugar X/Sugar Y indicates Sugar X/Sugar Y half-and-half medium.

To examine whether each monosaccharide was taken up or metabolized by *E. histolytica* trophozoites, we measured the remaining amounts of monosaccharides in 96-h culture media using HPLC. All monosaccharides had different retention times with single peaks, except that D-psicose appeared as two peaks for an unknown reason (Supplementary Figure S3). About 80% of D-glucose in D-glucose medium was uptaken by trophozoites, while the amount of D-galactose uptaken varied among the studies (Figure 7). This was because most trophozoites were detached from the vial walls and could not proliferate, but some remained on the wall and proliferated in an anchorage-dependent manner, since all cells could not be detached artificially while culturing the trophozoites. Therefore, variation in remaining D-galactose was present in all results from D-galactose containing media, including D-galactose, Glc/Gal, Gal/Fru, Gal/All and Gal/Psi media. Nevertheless, equal amounts of D-glucose and D-galactose were taken up by trophozoites in Glc/Gal medium: Glc (Glc/Gal) and Gal (Glc/Gal). Interestingly, the remaining D-glucose in Glc/Gal media was equivalent to that in D-glucose medium in 96-h culture, even though the amount of D-glucose in D-glucose medium was double at 0 h compared with that in Glc/Gal medium. D-Fructose in media was not decreased in D-fructose, Glc/Fru and Gal/Fru media, even after 96-h culture, indicating that D-fructose was not uptaken, metabolized or stored by *E. histolytica* trophozoites. D-Allose and D-psicose were also not uptaken, metabolized or stored by the trophozoites. D-Galactose was decreased after 96-h culture in Gal/Fru, Gal/All and Gal/Psi media, indicating that D-galactose was uptaken and metabolized or stored by the trophozoites. Uptake of D-glucose was higher in Glc/Fru, Glc/All and Glc/Psi media compared with that in Glc/Gal medium, suggesting that D-galactose inhibited or competed with D-glucose uptake by the trophozoites.

## 4 Discussion

*E. histolytica* lacks proteins of the tricarboxylic acid (TCA) cycle and electron transport chain because it does not have typical mitochondria. Therefore, the parasite utilizes glycolysis as the main pathway for energy production. However, trophozoites lost some of the genes in this pathway and obtained these by lateral gene transfer (LGT) (Loftus et al., 2005). Since the glycolysis pathway is vital for trophozoites, we examined whether monosaccharides including rare sugars affect their proliferation *in vitro*.

**FIGURE 8:**
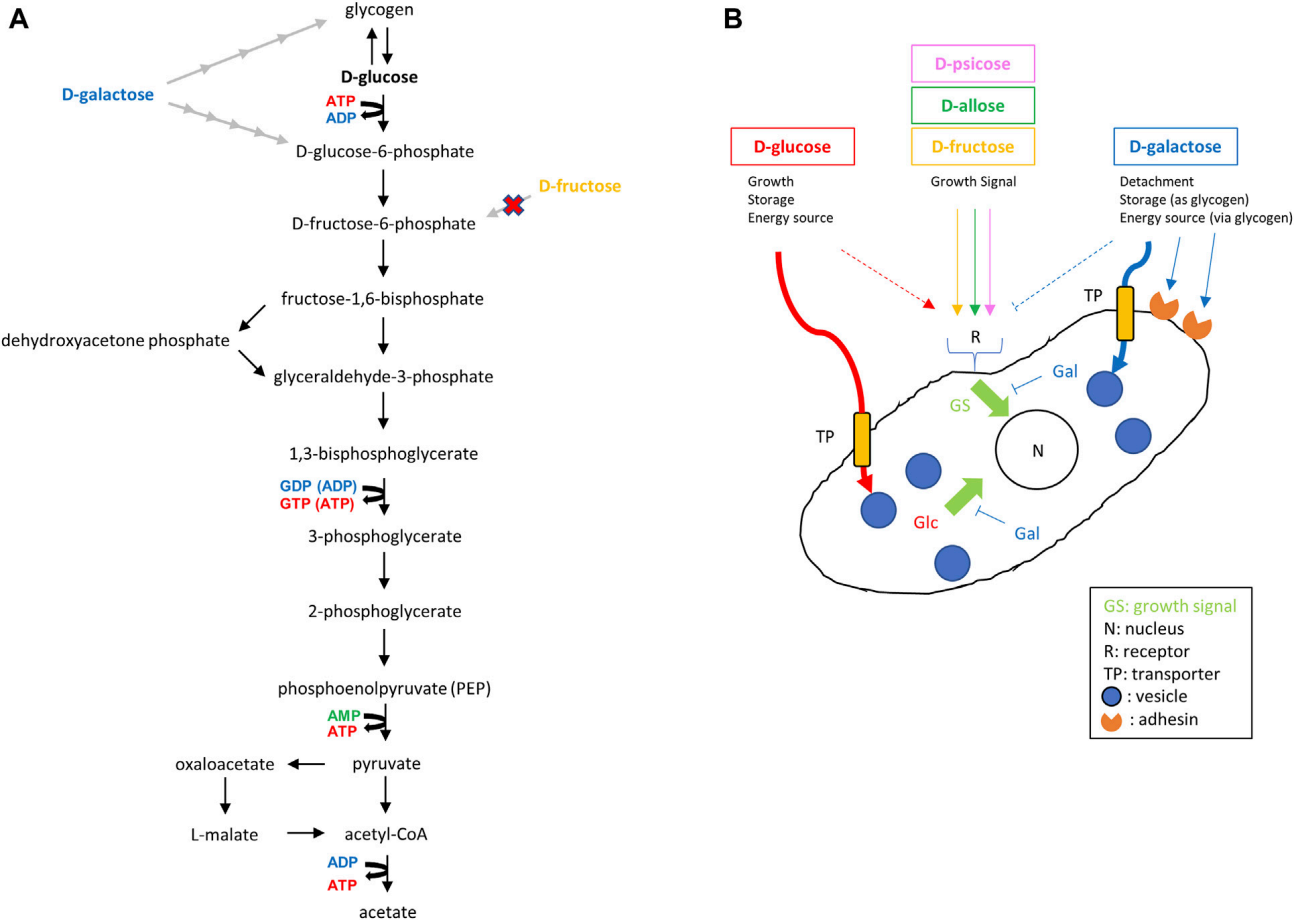
Predicted glycolysis pathway and effect of monosaccharides on proliferation of *E. histolytica* trophozoites. **(A)** Predicted glycolysis pathway in *E. histolytica* trophozoites (Reeves, 1984; Loftus et al., 2005; Jones and Ingram-Smith, 2014; Pineda et al., 2016). D-Galactose and D-fructose can be converted to D-glucose-6-phosphate and D-fructose-6-phosphate, respectively. However, D-fructose was not taken up by trophozoites (Figure 7). **(B)** Illustration of the predicted effects of monosaccharides from results obtained in this study. D-Fructose, D-allose and D-psicose do not enter trophozoites, but could enhance growth of trophozoites in the presence of D-glucose or D-galactose. This may indicate the presence of receptors transducing growth signals for those sugars.

As shown in Figure 8A, *E. histolytica* has a predicted glycolysis pathway with conversion of acetyl-CoA to acetate to produce additional ATP (Reeves et al., 1977; Loftus et al., 2005; Jones and Ingram-Smith, 2014; Pineda et al., 2016). Based on this pathway, *E. histolytica* trophozoites seem to be able to utilize D-galactose as an energy source for proliferation. However, trophozoites in D-galactose medium detached from the glass wall of vials and could not proliferate well in the current study. Galactose/*N*-acetylgalactosamine-inhibitable lectins of *E. histolytica* trophozoites may have roles for the attachment (Petri et al., 2002), but the result may indicate that other D-galactose-mediated mechanisms exist for the adherence of the trophozoites to glass surfaces. Furthermore, the size of the trophozoites became larger than in D-glucose medium, indicating that D-galactose was uptaken, converted and stored as glycogen, presumably in glycogen granules (Reeves, 1984). The size of *E. histolytica* trophozoites has varied among reports (Hoare, 1952; Freedman and Elsdon-Dew, 1958; Diamond and Clark, 1993; Ali and Nozaki, 2007; Aguilar-Díaz et al., 2010), which may be due to environmental effects (Freedman and Elsdon-Dew, 1958), but the exact reason is unknown (Hoare, 1952). The size of trophozoites in our study ranged from 15 to 40 μm depending on the culture conditions. The reasons for the size variation and the relationship with virulence are unclear, but our results indicate that monosaccharides in culture media can affect the size of the trophozoites.

Use of D-galactose for proliferation by trophozoites in the absence of D-glucose was also indirectly proven in our study by the gradual proliferation seen in Gal/Fru, Gal/All and Gal/Psi media. The growth observed in these media may be the same as that observed in Glc/Fru, Glc/All and Glc/Psi media with a time lag because conversion from D-galactose to glycogen or D-glucose-6-phosphate requires several steps. Such conversion also decreased the amount of D-galactose in the media, which allowed the trophozoites to reattach to the vial walls and permit proliferation in an anchorage-dependent manner. It remains unclear why trophozoites could not grow in Glc/Gal and D-galactose media, but this may suggest that D-galactose has a suppressive effect on trophozoite proliferation directly or indirectly by competing with D-glucose (Figure 8B). It may be because the transport systems for D-glucose and D-galactose are common and differ from that for D-fructose (Reeves, 1984). Our study suggests that D-fructose, D-allose and D-psicose are not well-transported in the cytosol or vesicles of the parasite because the size of the trophozoites in these media was smaller than that of trophozoites in D-glucose or D-galactose media, and D-fructose, D-allose or D-psicose was not uptaken, metabolized or stored in the trophozoites based on HPLC analysis. This may indicate that trophozoites survive by storing glycogen, which may be preferentially generated from D-glucose rather than D-galactose, while floating. This may be a strategy for trophozoites to survive and flourish because some detached trophozoites in D-glucose medium overgrown in culture are viable, and will attach and proliferate again after transfer to new flasks or tubes (data not shown). Delayed proliferation with low growth of trophozoites in D-galactose medium has been mentioned previously (Reeves, 1984). Such growth was not observed over the period we examined, but it is possible that trophozoites in Glc/Gal or D-galactose media would start to grow again in a prolonged culture period.

Another way to generate ATP through glycolysis is to use D-fructose instead of D-glucose (Figure 8A). Based on the genome of *E. histolytica*, it is likely that the enzyme(s) to convert D-fructose to D-frutose-6P was acquired by LGT from bacteria (Loftus et al., 2005). In our study, trophozoites grew within 24 h in culture, but the number of viable trophozoites decreased thereafter. This result agrees with the lower oxygen uptake seen for *E. histolytica* trophozoites in D-fructose medium compared with that in D-glucose or D-galactose medium (Weinbach and Diamond, 1974), and the observation that crude glucokinase or recombinant hexokinases of *E. histolytica* cannot utilize D-fructose as a substrate (Reeves et al., 1967; Kroschewski et al., 2000). Moreover, HPLC analyses showed that D-fructose was not uptaken, metabolized or stored by *E. histolytica* trophozoites (Figure 7). Therefore, even if enzymes that metabolize D-fructose are present in the cytoplasm, the trophozoites cannot utilize D-fructose as an energy source. Recently, *E. histolytica* trophozoites adapted in fructose culture were found to remain viable for at least 1 year (Matt and Duchêne, 2015). The authors suggested that this may be possible because a gene product, similar to bacterial fructokinase, of the parasite acquired by LGT can phosphorylate fructose. We could not reproduce this result, which may be due to the frequency of changing media. It may vary among cattle breeds, but adult bovine serum contains 100–300 mg/dL glucose (Yu et al., 2013), which corresponds to 0.8–2.5 mM glucose in TYI-S-33 medium. This may allow trophozoites to survive even in fructose medium after a change to fresh medium in the short term (e.g., every 24 h). Reeves (1984) also suggested that components of TYI-S-33 medium, without added sugar, contribute sufficient glucose to allow survival of serial transplants of small numbers of amoebae. Further studies are needed to examine this issue.

D-Allose and D-psicose media had comparable effects to those in medium without sugars. The precise mechanisms remain unclear, but reactive oxygen species were produced in *E. histolytica* trophozoites cultured in those media, suggesting that oxidative stress had a role in the effects (Supplementary Figure S4). This may indicate that these rare sugars alone can be used as sweeteners with anti-amoebic functions. However, these sugars somewhat enhanced the proliferation of *E. histolytica* trophozoites caused by D-glucose and allowed trophozoites to grow gradually with D-galactose. Our preliminary studies suggest that higher concentration than 45 mM of rare sugars, in total 51.4 mM of sugars adjusted with D-glucose, may be effective for suppression of the growth of *E. histolytica* trophozoites (Supplementary Figure S6). However, the content of nutrients in the intestine, the habitat of the trophozoites, is more complex than that used in the present *in vitro* study. The absorption of glucose takes place in the small intestinal epithelium (Chen et al., 2016) and the large intestine, which is the site of *E. histolytica* infection, has very low levels of glucose (Tovy et al., 2011). In contrast, 20%–30% of orally administered D-psicose, which alone can suppress the growth of trophozoites, will reside in the large intestine (Whistler et al., 1974; Tsukamoto et al., 2014) indicating that it has a potential as an anti-amoebic substance *in vivo*. Therefore, there is a need for further studies of use of rare sugars as anti-amoebic substances. A concern with rare sugars is their growth inhibitory effects, which have been observed for cancer cells and *C. elegans* (Sato et al., 2008; Sakoguchi et al., 2016a; Sakoguchi et al., 2016b; Noguchi et al., 2016). Adults and children under 5 years old who are malnourished can also be infected by *E. histolytica* (Fauziah et al., 2022), and there is a need to examine the adverse effects of rare sugars on growth retardation of children, even though some rare sugars are already used in food products (Ahmed et al., 2022; Smith et al., 2022). After clarification of these issues, rare sugars and derivatives may also be useful for treatment of other infectious diseases caused by amitochondrial protist pathogens such as *Giardia* spp. and *Trichomonas* spp.

## Data Availability

The original contributions presented in the study are included in the article/Supplementary Material, further inquiries can be directed to the corresponding author.
