# Supplementary figures and images for: Effects of monosaccharides including rare sugars on proliferation of Entamoeba histolytica trophozoites in vitro
Source: Front Mol Biosci. 2023 Dec 8;10:1288470. doi: 10.3389/fmolb.2023.1288470 (PMC10739481; doi:10.3389/fmolb.2023.1288470)

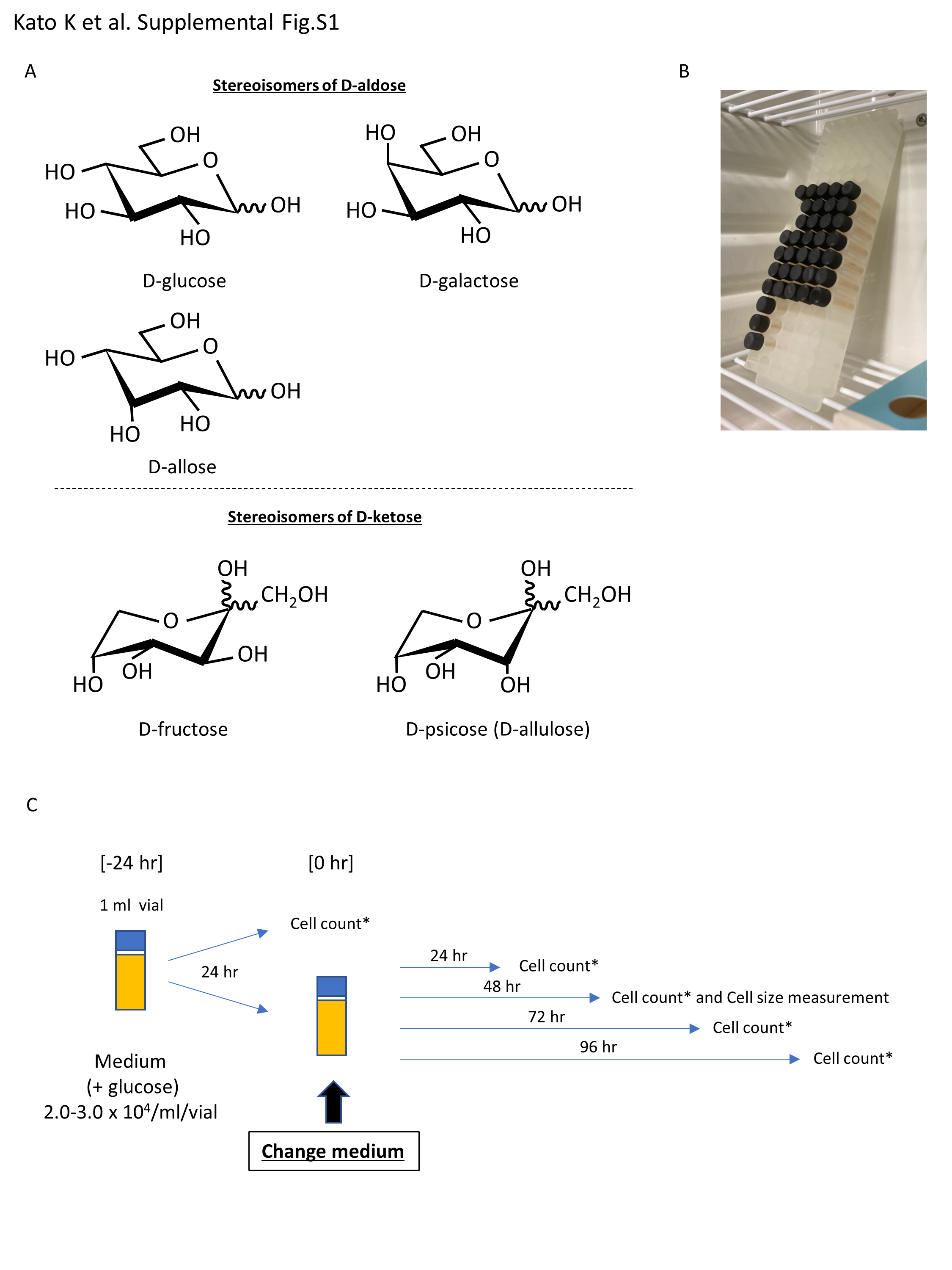

Supplement: Supplementary file 1 [file DataSheet1.zip › Supplementary Figures/Supplementary Figure 1.TIF]

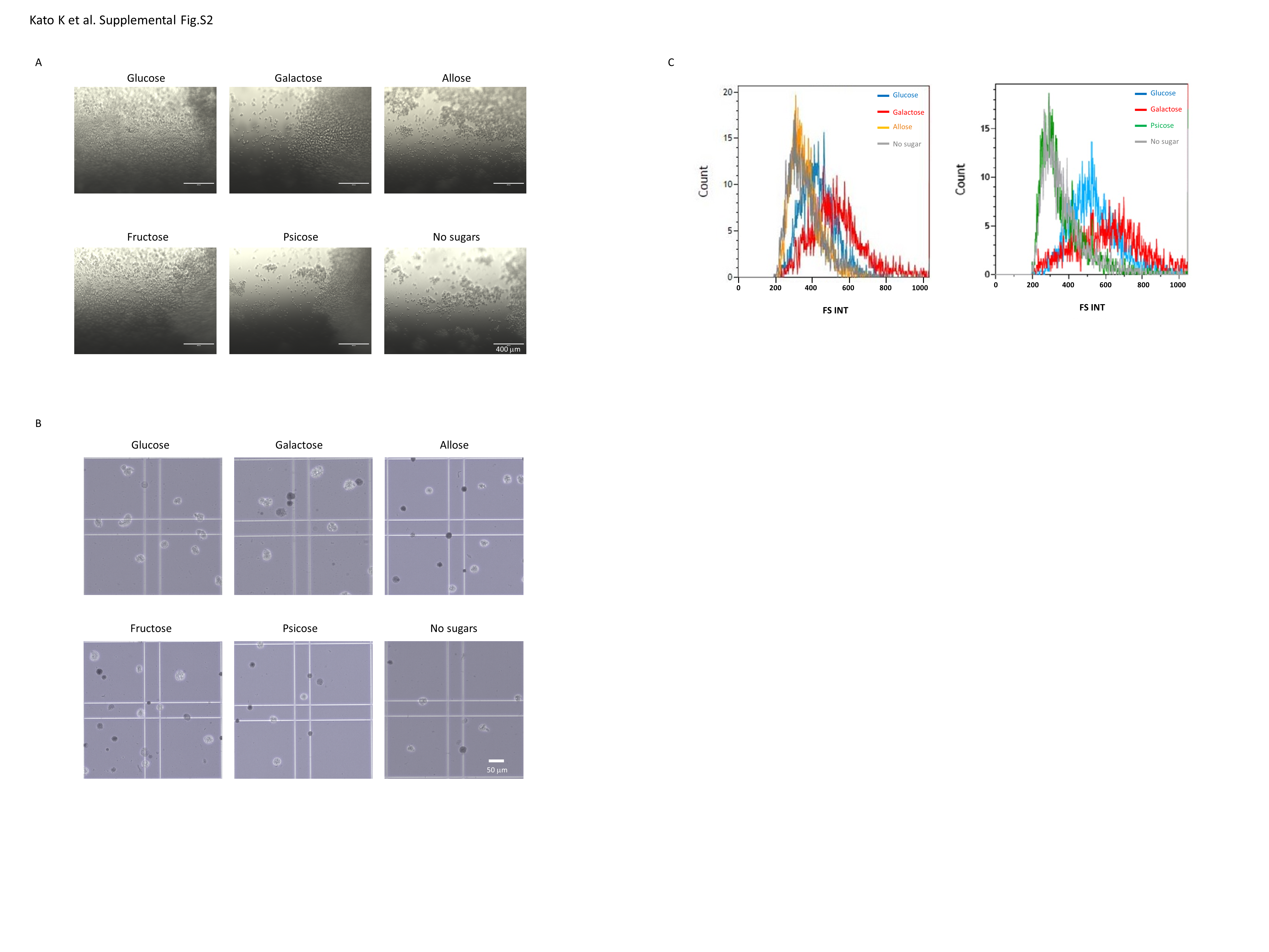

Supplement: Supplementary file 1 [file DataSheet1.zip › Supplementary Figures/Supplementary Figure 2.TIF]

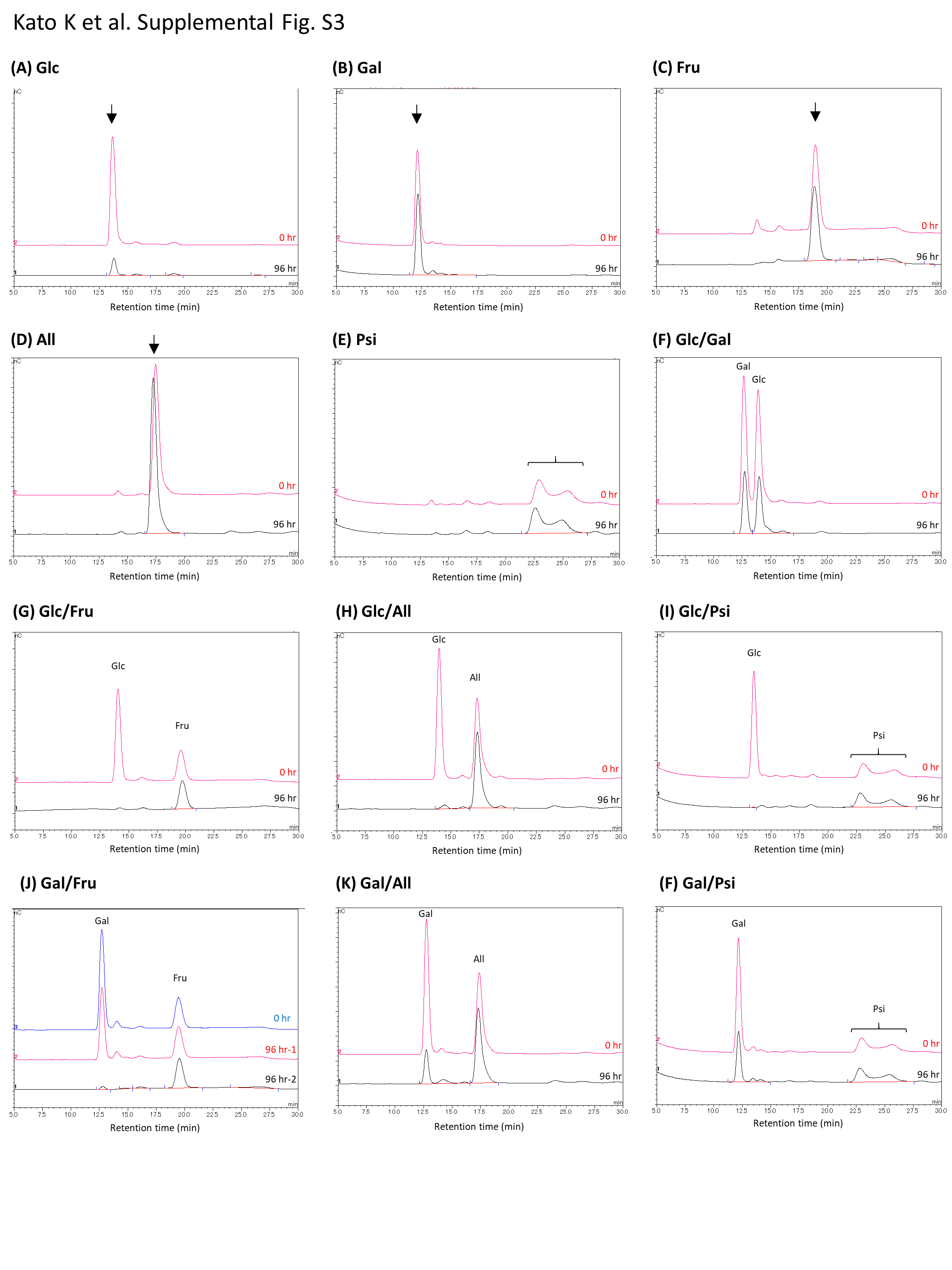

Supplement: Supplementary file 1 [file DataSheet1.zip › Supplementary Figures/Supplementary Figure 3.TIF]

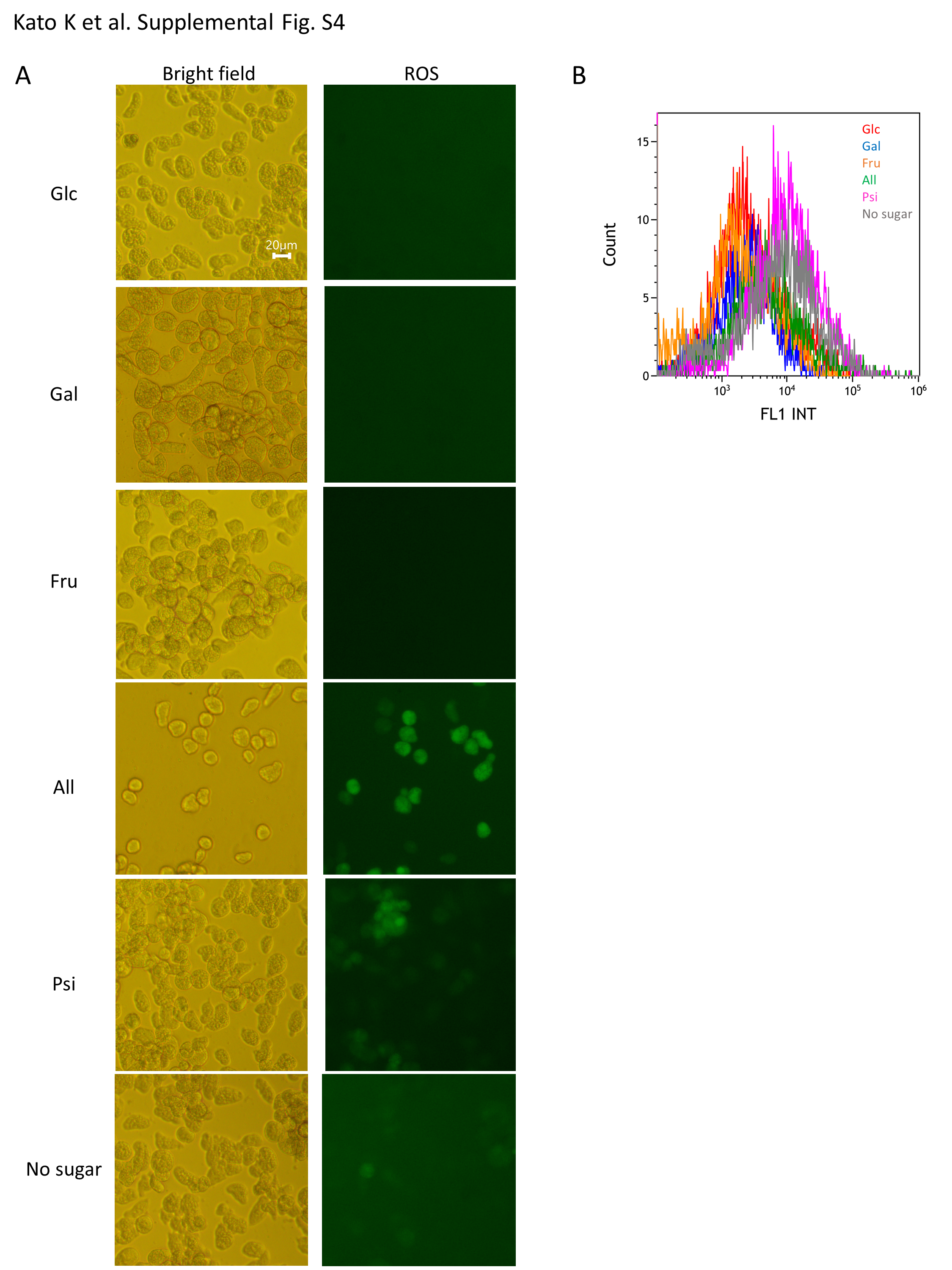

Supplement: Supplementary file 1 [file DataSheet1.zip › Supplementary Figures/Supplementary Figure 4.TIF]

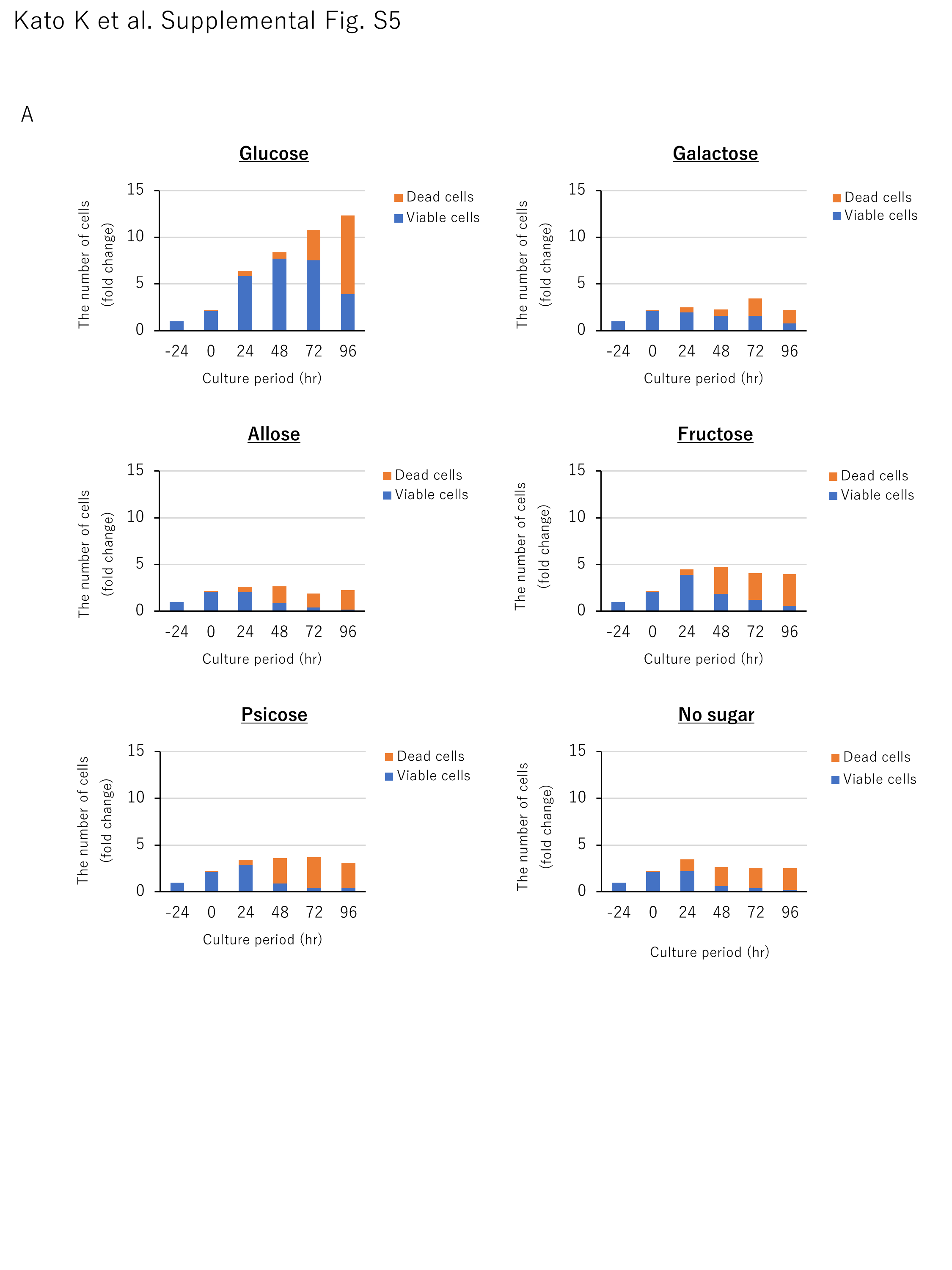

Supplement: Supplementary file 1 [file DataSheet1.zip › Supplementary Figures/Supplementary Figure 5a.TIF]

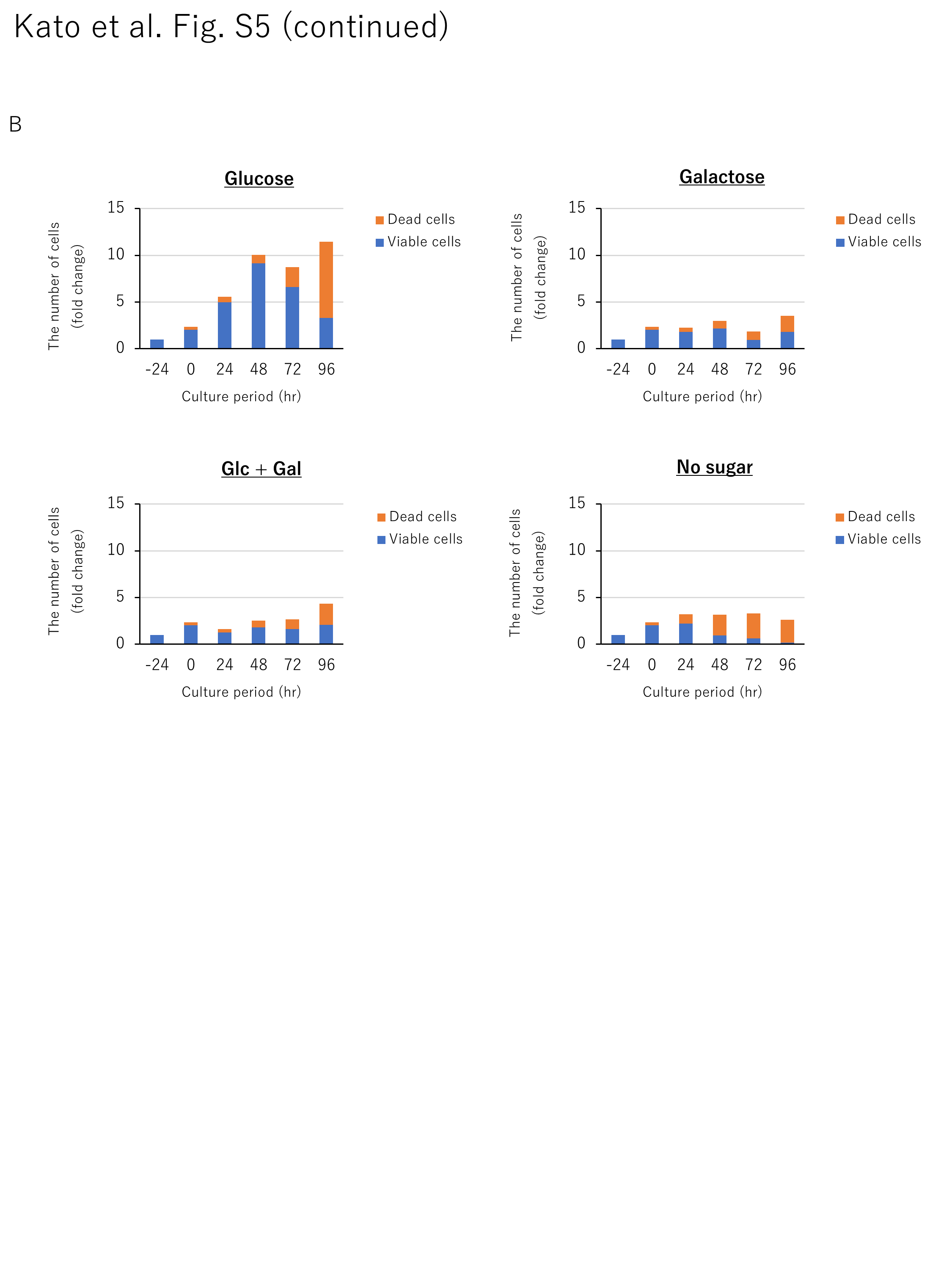

Supplement: Supplementary file 1 [file DataSheet1.zip › Supplementary Figures/Supplementary Figure 5b.TIF]

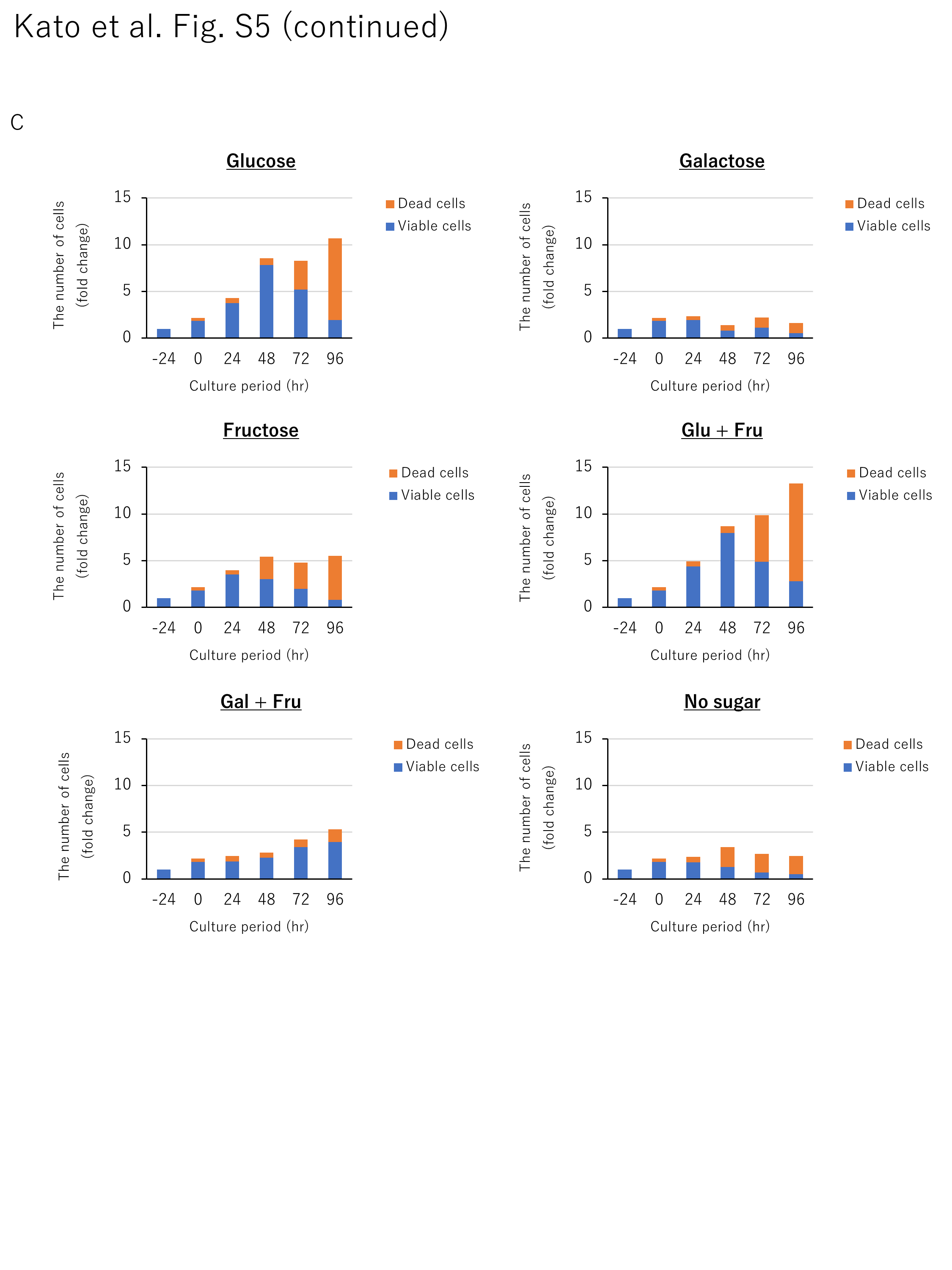

Supplement: Supplementary file 1 [file DataSheet1.zip › Supplementary Figures/Supplementary Figure 5c.TIF]

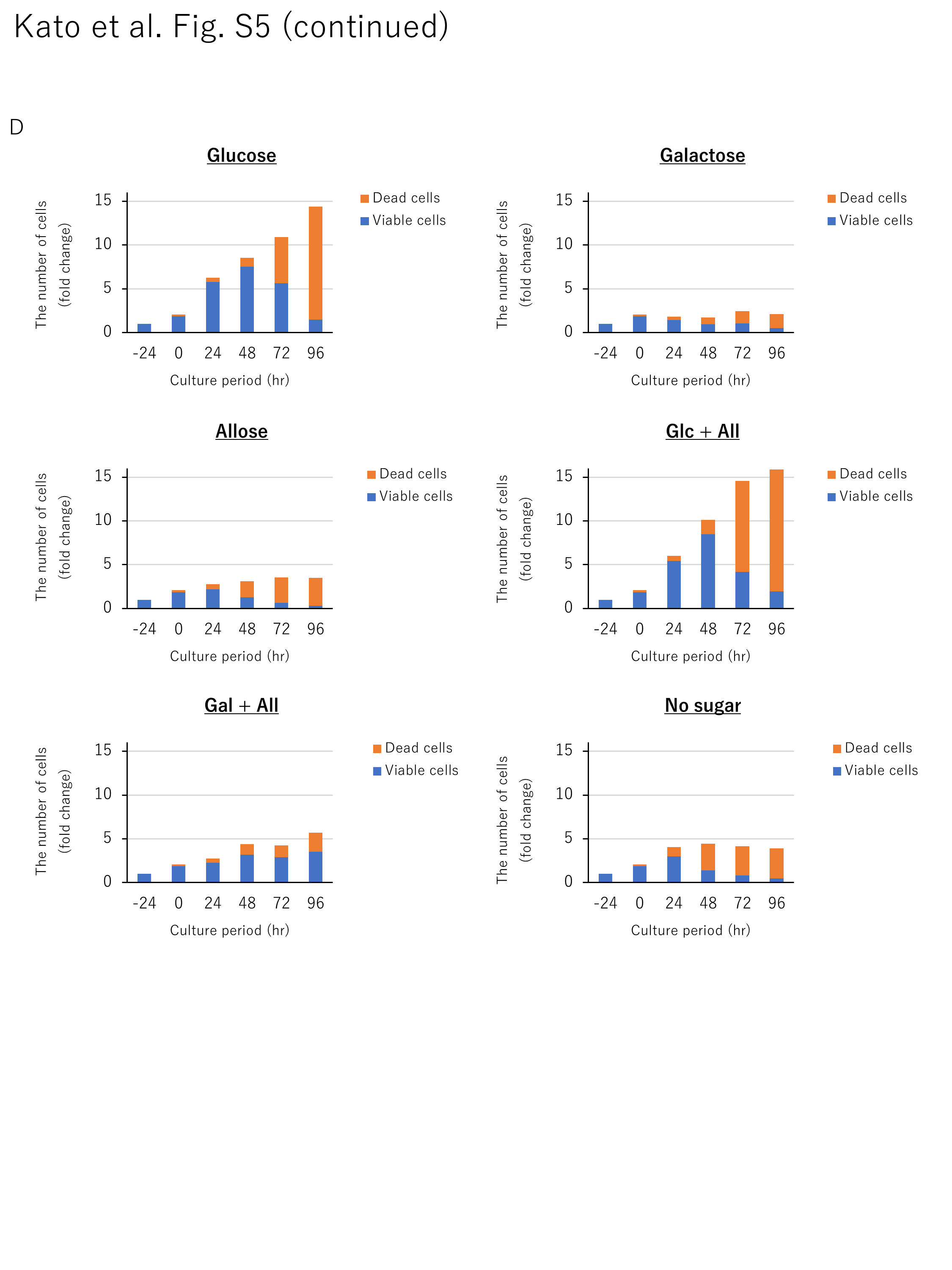

Supplement: Supplementary file 1 [file DataSheet1.zip › Supplementary Figures/Supplementary Figure 5d.TIF]

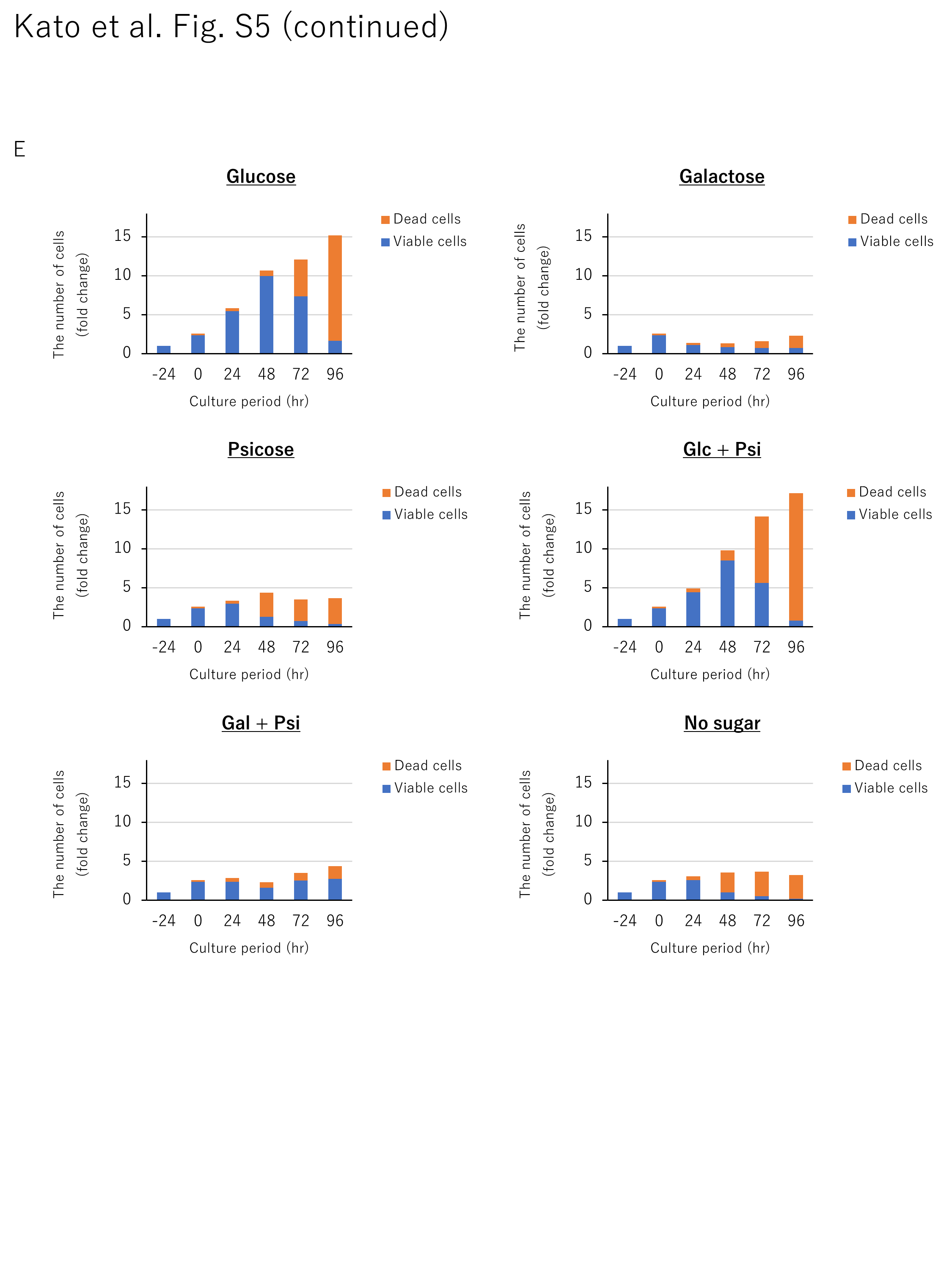

Supplement: Supplementary file 1 [file DataSheet1.zip › Supplementary Figures/Supplementary Figure 5e.TIF]

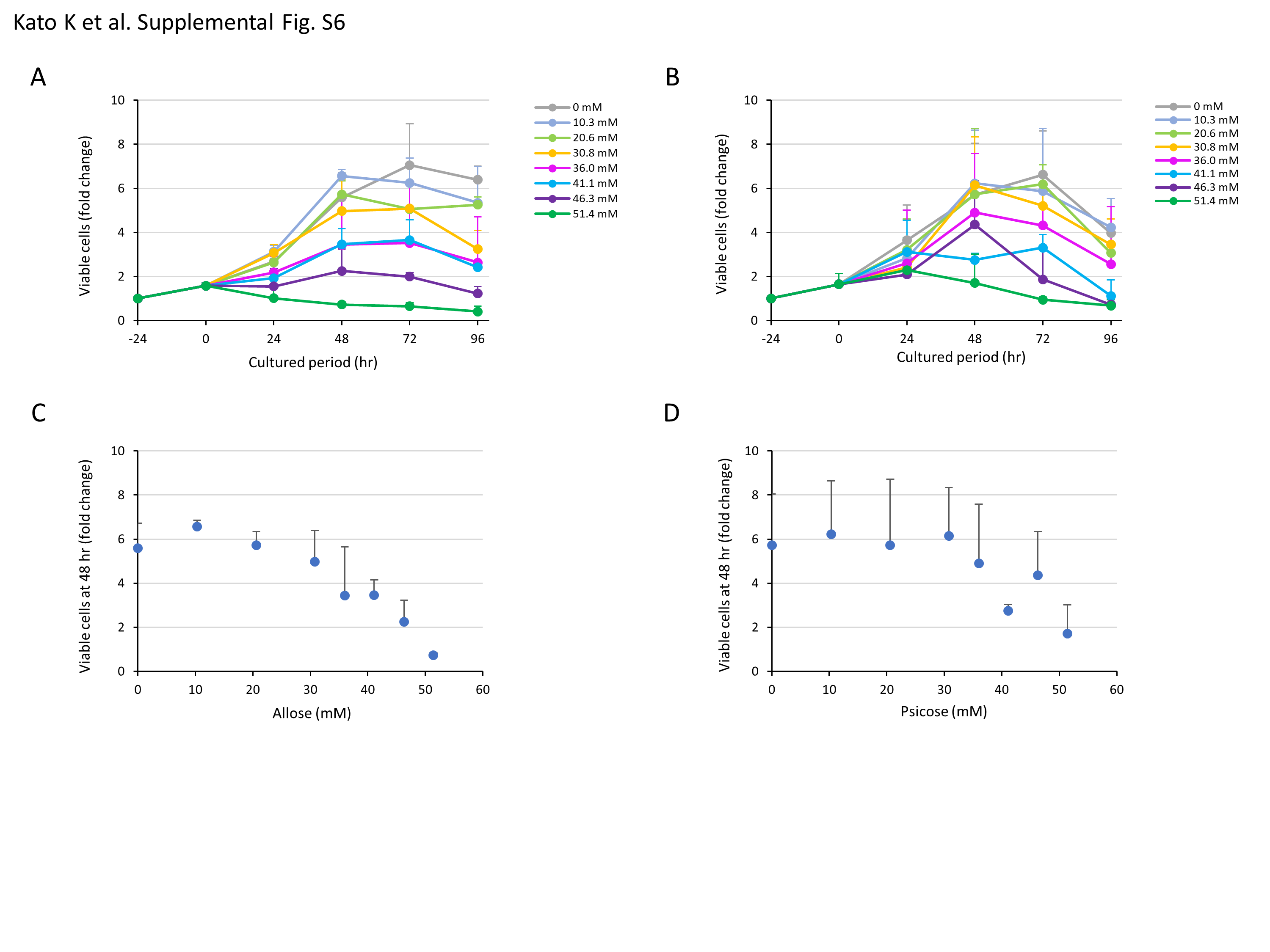

Supplement: Supplementary file 1 [file DataSheet1.zip › Supplementary Figures/Supplementary Figure 6.TIF]
